# Subjective and Objective Assessments of Post-traumatic Olfactory Dysfunction

**DOI:** 10.3389/fneur.2020.00970

**Published:** 2020-08-26

**Authors:** Nattakarn Limphaibool, Piotr Iwanowski, Wojciech Kozubski, Teodor Swidziński, Anna Frankowska, Ilona Kamińska, Kamila Linkowska-Swidzińska, Alicja Sekula, Piotr Swidziński, Zofia Maciejewska-Szaniec, Barbara Maciejewska

**Affiliations:** ^1^Department of Neurology, Poznan University of Medical Sciences, Poznań, Poland; ^2^Department of Biophysics, Poznan University of Medical Sciences, Poznań, Poland; ^3^Department of Medical Law, Organization and Management in Healthcare, Poznań University of Medical Sciences, Poznań, Poland; ^4^Department of Phoniatrics and Audiology, Poznan University of Medical Sciences, Poznań, Poland; ^5^Department of Conservative Dentistry and Endodontics, Poznan University of Medical Sciences, Poznań, Poland; ^6^Department of Temporomandibular Joint Disorders, Poznan University of Medical Sciences, Poznan, Poland

**Keywords:** olfactometry, olfactory dysfunction, olfactory evaluation, olfactory event-related potentials, traumatic brain injury

## Abstract

**Introduction:** Traumatic brain injuries are the most common cause of olfactory dysfunction. Deficits in olfaction may be conductive or neurosensory in nature, with varying degrees of impairment resulting in a diminished quality of life and an increased risk for personal injury among patients. The aim of this research is to evaluate the results of the subjective and objective quantitative examinations of olfactory function in a group of patients with post-traumatic anosmia in order to predict its value in identifying olfactory deficits in clinical practice.

**Materials and Methods:** The present study included 38 patients who reported anosmia or hyposmia caused by a traumatic head injury, and a group of 31 age- and sex-matched controls without olfactory dysfunction or prior history of head injury. The comparison of odor perception and identification of two oils (mint and anise) was assessed with the use of blast olfactometry with cortical olfactory event-related potentials.

**Results:** Subjective olfactory tests revealed anosmia or hyposmia in 94% of patients with head injury-related olfactory dysfunction. Objective tests revealed olfactory event-related potentials from cranial nerve I produced by the stimulation with both mint and anise in 20 patients (52.6%). Olfactory event-related potentials from cranial nerve V produced by the stimulation with mint were registered in 26 patients (68.4%). The lack of any responses, from both cranial nerve I and V, was found in 12 patients (32% of cases).

**Conclusions:** Findings from our study indicate the application of both subjective and objective examinations in the evaluation of patients with olfactory impairment. In the diagnosis of post-traumatic anosmia or hyposmia, objective examinations are particularly useful when the patients' level of cognition may be impaired or when subjects may be exaggerating their olfactory defects for a secondary gain. The diagnosis of damage to the olfactory system, specifically in the receptive part of the olfactory pathway, can be established in patients who showed reduced amplitudes or absent cortical responses in addition to absent odor identification and perception threshold in the subjective examination.

## Introduction

Olfactory dysfunction is commonly reported in the aftermath of a traumatic brain injury (TBI), through mechanisms including a direct damage to the olfactory nerve fibers at the cribriform plate, injury to the sinonasal tract and hemorrhage within the olfactory cortex (1). Deficits in olfaction may be conductive or neurosensory in nature, with varying degrees of impairment depending on factors including the severity of head trauma, site and nature of injury, and duration of post-traumatic amnesia (2, 3). Patients with olfactory impairment may experience a diminished quality of life (QoL) and an increased risk for personal injury. The utility of quantitative identification olfactometry in identifying central olfactory impairment, a highly specific marker for structural neurologic injury has been supported by recent research (4). An investigation of olfactory deficit and its impact on the patient is crucial for follow-up treatment and counseling (1).

Verification of olfactory impairment and the assessment of its severity requires thorough olfactory testing. Subjective response to olfactory challenge tests are commonly used in clinical practice in the evaluation of patients with neurological disorders. However, research indicates that it is not quantifiable, is easily feigned by malingerers, and is problematic for studies requiring follow-up investigations. Its limited use in patients with cognitive impairment makes it particularly unreliable in a significant number of patients with a history of TBI (5, 6). Objective examination with event-related potentials, on the other hand, may be useful in identifying olfactory defects. However, these techniques are complex, time-consuming, costly, and not routinely performed in the clinical practice (7).

The aim of the present study is to evaluate the results of the subjective and objective quantitative examinations of olfactory function in a group of patients with post-traumatic anosmia in order to predict its value in identifying olfactory deficits in clinical practice. The analysis includes the comparison between odor perception and identification thresholds with the use of blast olfactometry and the amplitude of recorded olfactory event-related potentials (OERPs) as subjective and objective methods of olfactory testing, respectively. Additional factors including the degree of olfactory impairment in relation to the mechanism of head injury and the impact of diminished olfaction to patient's QoL and professional work were evaluated.

## Materials and Methods

### Study Population

The present study consists of a study group of 38 patients (16 females and 22 males; mean age 45 ± 16.1 years) with post-traumatic olfactory dysfunction, who have been evaluated in the Department of Neurology, Poznan University of Medical Sciences, between 2007 and 2017. The control group consists of 31 sex- and age-matched healthy subjects (14 females and 17 males; mean age: 51 ± 14.6 years) without signs or symptoms of olfactory dysfunction or neurological impairment. The exclusion criteria for the study included patients with impaired cognition, poor cooperation, post-traumatic aphasia, and patients with a medical history of neurodegenerative disorders. Patients who suffered from injuries caused by contact sports were also excluded from the study as contact sports increase the risk of nasal bones fracture and damage to the olfactory region in the nasal cavity. All patients underwent detailed neurological examination and mental assessment. All patients were alert, well-cooperative, and had good comprehension. No mental illness or impaired cognition was suspected (all patients had normal outcomes of Mimi-Mental State Examination (MMSE) A careful medical history was obtained from all participants with regards to the etiologies of head trauma and accompanying neurological deficits.

The first part of the study included a short questionnaire, in which respondents answered (1) whether their olfactory dysfunction significantly aggravated their QoL, and (2) whether their olfactory dysfunction have had an impact on their professional work. The second part of the study included the subjective and objective examinations of olfaction which were performed within 4 months to 2 years subsequent to the subjects' head injury.

During the olfactory examinations, the subjects were secluded from other sensory stimuli, such as sudden changes in light, sound, or temperature, while maintaining a stress-free comfortable position. A continuous sound signal in the form of white noise (75 dB) were administered via headphones to the tested subjects. The intensity of this noise was selected during the apparatus tests, at a level which sufficiently masks the ambient sounds such as those from the mechanical elements of the odor dispenser to prevent accidental cortical auditory responses. The instruction was simple: please hold up your right arm if you can smell anything.

### Fragrances Used in Olfactometric Test

The odors employed in the olfactometric testing includes mint (100% natural mentha piperita oil) and anise (100% natural Illicium Verum Seed Oil) at the temperature of 21 ± 1 degrees Celsius. Anise oil stimulated olfactory nerve endings whereas mint oil stimulated both the olfactory and trigeminal nerve endings in the nasal mucosal tissue (8, 9). Fragrances are often exchanged, allowing constant concentrations of particles within the air supplied. The bottles were tightly closed with stoppers in which there were 2 openings through which the glass tubes pass. Plastic drains are connected to the tubes. A metal nasal terminal and a clamp which regulates air flow were placed on one drain and a syringe was attached to the second drain. The glass containers were made of dark glass, making it impossible for the subject to assess the interior contents. The subjects' responses to olfactory stimuli were recorded, according to the predetermined methodology of Poznan University of Medical Sciences.

### Subjective Olfactory Evaluation

The examinations of olfactory function were performed with blast (Elsberg-Levy) olfactometry, a popular method of olfactory threshold measurement. This involves the introduction of a stream of air with a well-defined volume containing fragrance molecules into the nasal cavity. The nasal tip is placed in the vestibule of the nose, with the long axis of the tip forming a 45° angle with the horizontal plane. The patient simultaneously closes the other nasal passage with their finger by pressing on the nostrils and momentarily holding their breath. A clamp is placed on the tube which supplies air to the nasal cavity.

A specific volume of air was delivered to the container, then the clamp on the second drain was released, creating a blast of air mixed with the vapors of the fragrance that reaches the olfactory region. One cm^3^ of air was initially administered to the nasal cavity, and increasing volume of 1–2 cm^3^ were gradually administered until the subject reports an odor sensation. The test continued until the patient were able to identify the perceived odor and the results in cm^3^ were recorded. To avoid the phenomenon of olfactory fatigue, subsequent blasts were uniformly delivered to one nasal cavity no more frequently than every 30 s, and to the other nasal cavity after 2 min.

Two values were achieved as a result of the test: (1) The olfactory perception threshold, defined as the minimum volume of air supplied to the nasal cavity at which the subject reported an olfactory sensation. (2) The olfactory identification threshold, defined as the volume of air at which the subject can accurately describe the type of fragrance.

The physiological values of odor perception are 2–6 and 2–14 cm^3^ for mint and anise, respectively. If no concentration induced perception, the patient was considered anosmic for the odor in question. The physiological values for the odor identification threshold are 3–17 and 5–21 cm^3^ for mint and anise, respectively. If identification was correct, the identification threshold coincided with the perception threshold. If the identification could not be made or is falsely stated, the increasing stimulus method was implemented. The lowest concentration at which the odor was correctly identified constituted its identification threshold. If no identification was obtained even at the highest concentration of stimuli, the patient was considered not to recognized the odor in question.

### Objective Olfactory Evaluation

OERPs are a valid electrophysiological technique which observes changes in olfactory function in an objective manner. The recording device for cortical evoked potentials (ERA 2250 firmy Madsen Electronics) and odor stimulator according to the abovementioned Elsberg Levy method were used in the objective olfactory evaluation. The electrode position was based on the 10–20 international system for electrode placement. Due to the relatively rapid adaptation of the olfactory system to the administered stimuli, the technique of summing and averaging in this test includes only several single responses. Studies have used OERPs to evaluate olfactory function in diverse medical conditions including multiple sclerosis, Alzheimer's disease, and cognitive impairment (10, 11).

### Statistical Analysis

The statistical analysis of all collected data was carried out using the Statistica v.10. The analysis was performed by a professional statistician from the Department of Computer Science and Statistics, UMP. The methods included statistical description, analysis of correlation and statistical conclusion. Kruskal-Wallis one-way ANOVA was used to evaluate the correlation between olfactory impairment and the etiologies of head injury and Wilcoxon difference test were used to compare the results of subjective and objective olfactory examinations in the study. For all statistical tests, a *p* < 0.05 was considered as significance level.

### Ethical Considerations

The study was conducted in accordance with the Declaration of Helsinki and was approved by the Bioethics Committee of Poznan University of Medical Sciences (n. 1097/16). All subjects gave written informed consent before any study-related procedures were performed.

## Results

### Patient Demographic

Etiologies of head trauma among patients in the studied group included motor vehicle accidents (16 patients, 42%), domestic falls (16 patients, 42%), and physical assaults (6 patients, 16%). CT/MR examinations revealed facial fractures in 20 patients (53%) and cerebral contusion in 18 cases (47%). Eighteen patients (47%) suffered concussive symptoms and 4 patients (11%) were diagnosed with auditory dysfunction (Table 1). No significant correlation was found between olfactory impairment and the etiologies of head injury (Kruskal-Wallis one-way ANOVA *p* > 0.05), or the presence of concussion in patients (*p* > 0.05). Seventy six percent of patients reported lowered QoL and 58% reported a significant impact on their professional work as a direct result of olfactory dysfunction.

**Table 1:** Patient demographic.

	**Patients (*n* = 38)**	**Controls (*n* = 31)**
Age, years	45 ± 16.1	51 ± 14.6
Females, *n* (%)	16 (42)	14 (45)
Etiologies of head trauma (n)	Motor vehicle accidents (16)	
	Domestic falls (16)	
	Physical assaults (6)	

### Olfactory Evaluation

Olfactory perception is a reaction to the stimulation of cranial nerve (CN) I and odor identification is a reaction to both CN I and CNV stimulation. Impaired olfactory perception with preserved identification suggests peripheral damage (i.e., olfactory nerve damage). Abnormal results on both tests may reflect pathology in the central olfactory pathways (12). Results from the subjective examinations of olfaction revealed that olfactory perception threshold was not achieved in 25 patients for mint (remaining 10 patients obtained perception threshold with increased volume (cm^3^) of stimuli, 3 patients within the normal range) and 26 patients for anise (remaining 9 patients obtained perception threshold with increased stimuli, 3 patients within the normal range). The olfactory identification threshold was not obtained in 36 patients (94.4%) (remaining 2 patients reported obtained identification threshold with increased stimuli).

**Figure 1:**
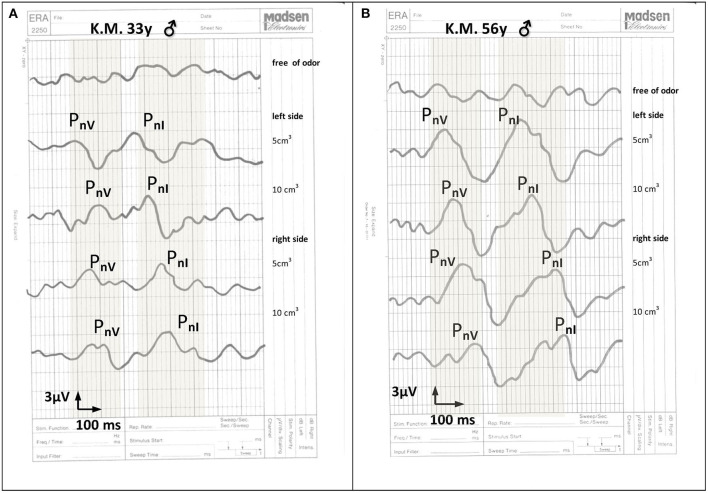
*The recording of cortical olfactory potentials to the stimulation with 5 and 10 cm^3^ mint essential oil: **(A)** in a 33-year old man 6 months after a head injury, **(B)** in a health 56-year-old man without olfactory dysfunction. The normal range for potentials PnI and pnV is marked in gray. Recorded cortical olfactory potentials after a head injury include latency periods within normal limits with lowered response amplitudes. PnI = responses to stimuli to nerve I endings. PnV = responses to stimuli to nerve V endings.

Objective examinations of olfaction revealed OERPs from CN I to the stimulation with both mint and anise in 20 patients (53%). OERPs from CNV to the stimulation of mint alone was observed in 26 patients (68%). Twelve patients (32%) showed no responses to either CNI or CNV. When OERPs PnI and PnV were present, latency periods were within the normal limits. The amplitudes of recorded electrophysiological responses were reduced in 20 patients 236 (below 15 μV) and normal in 6 patients (above 16 μV). Figures 1A,B presents the recording 237 of OERPs in examined individuals.

### Comparison of Subjective and Objective Tests

The correlation between subjective (olfactory perception and identification threshold) and objective examination (assessment of latency periods and amplitudes of potentials PnI and PnV) was analyzed. Tables 2, 3 presents the results of the difference between these two examinations. A statistically significant difference was found between the assessment of latency periods of responses (objective examination) and the olfactory perception and identification threshold (subjective examination) (Table 4). No statistically significant differences were noted between the assessment of amplitudes of responses (objective examination) and the olfactory perception and identification thresholds (subjective examination) (Table 5).

**Table 2A:** Results of blast olfactometry in patients with post-traumatic olfactory dysfunction according to Elsberg-Levy.

	**Odor perception [cm**^****3****^**]**		**Odor identification [cm**^****3****^**]**	
	**Anise**	**Mint**	**Anise**	**Mint**
Study group^*^	*n* = 11	*n* = 11	*n* = 2	*n* = 2
Mean	22	18	Absent results	Absent results
Minimum	5	3	Absent results	Absent results
Maximum	50	45	Absent results	Absent results
Standard deviation	16	14	Absent results	Absent results

* *No odor perception n = 27, no odor identification n = 36*.

**Table 2B:** Results of blast olfactometry in the control group according to Elsberg-Levy.

	**Odor perception [cm**^****3****^**]**		**Odor identification [cm**^****3****^**]**	
	**Anise**	**Mint**	**Anise**	**Mint**
Control group	*n* = 31	*n* = 31	*n* = 31	*n* = 31
Mean	8	5	12	10
Minimum	2	2	5	3
Maximum	14	6	21	17
Standard deviation	3	3	7	6

**Table 3A:** Results of Olfactory (chemosensory) event-related potentials (OERPs) in patients with post-traumatic olfactory dysfunction.

**OERPs**	**Latency [ms]**			**Amplitude [μV]**		
	**Anise**	**Mint**	**Mint**	**Anise**	**Mint**	**Mint**
Results (*n* = 26)^*^	Pn-I	Pn-I	Pn-V	Pn-I	Pn-I	Pn-V
Mean	610	615	310	23	19	30
Minimum	460	470	210	11	10	18
Maximum	690	700	400	45	48	50
Standard deviation	95	100	90	10	12	11

* *OERPs have not been recorded in 12 patients*.

**Table 3B:** Results of Olfactory (chemosensory) event-related potentials (OERPs) in the control group.

**OERPs**	**Latency [ms]**	**Amplitude [μV]**				
	**Anise**	**Mint**	**Mint**	**Anise**	**Mint**	**Mint**
Control group (*n* = 31)	Pn-I	Pn-I	Pn-V	Pn-I	Pn-I	Pn-V
Mean	602	595	320	22	18	35
Minimum	450	460	200	15	14	20
Maximum	710	700	410	40	42	50
Standard deviation	75	70	51	7	8	10

**Table 4:** Results of significance of differences in subjective and objective examinations (SE vs. OE) for latency periods of potentials PnI and PnV.

**Wilcoxon difference test**	**Significance level mint**	**Significance level anise**
OE for latency P_N1_ vs. SE	0.00055	0.00050
OE for latency P_NV_ vs. SE	0,00740	–

**Table 5:** Statistical results of the significance level of differences in subjective vs. objective examinations (SE vs. OE) for the amplitude of potentials Pn1 and PnV.

**Wilcoxon difference test**	**Significance level mint**	**Significance level anise**
OE for amplitude P_N1_ vs. SE	0.3258	0.1950
OE for amplitude P_NV_ vs. SE	0.0587	–

The latency periods of recorded OERPs were not increased in patients with hyposmia. The reduction in amplitude of OERPs, however, is correlated to the diagnosed cases of hyposmia. Results from the present study shows that the average latency periods (ms) of potentials PnI to anise and mint were 610 ± 95 (control group: 602 ± 75) and 615 ± 100 (control group: 595 ± 70), respectively. The average latency period of PnV to mint was 310 ± 90 (control group: 320 ± 51). The average amplitudes (μV) of potentials PnI to anise and mint among study subjects were 23 ± 10 (control group: 22 ± 7), and 19 ± 12 (control group: 18 ± 8), respectively. The average amplitudes of potentials PnV to mint was 30 ± 11 (control group: 35 ± 10).

Damage to the perceptive olfactory system can be identified when there is a reduction in amplitude or absence of OERPs in addition to impaired olfactory perception and identification in the subjective examination. On the other hand, cases with recorded OERPs but absent perception and identification olfactory threshold were suspected of feigning anosmia or suffering from cognitive dysfunction or stimulation connected with the compensation processes or evidence of regeneration processes of the olfactory system.

### Results From Control Subjects

No control subjects declared any olfactory disorder on physical examination. Perception thresholds were of normal range with both tested odors perceived at the lowest concentration. Identification thresholds were likewise of normal range, with both odors easily identified even at the lowest concentration. Objective examinations revealed OERPs from both CNI and CNV to stimuli and latency periods were within normal limits.

## Discussion

The present study examined the results of the subjective and objective testing of olfactory function in patients who reported impaired sense of smell after head trauma. The quantitative methods for evaluating olfactory function was the assessment of olfactory perception and identification threshold (a subjective examination), and an assessment of latency periods and amplitudes of OERPs (an objective examination). The main findings demonstrated by this study are:

(1) The use of both subjective and objective examinations may help to verify, assess and locate the damage to the olfactory system.

(2) Objectives examinations are useful in post-traumatic situations in which the patients' level of cognition may be impaired or when subjects may be exaggerating their olfactory defects for a secondary gain.

### Pathomechanisms of Post-traumatic Olfactory Dysfunction

Post-traumatic olfactory dysfunction may present secondary to direct damage to the olfactory nerve fibers at the cribriform plate, sinonasal tract disruption, and focal contusion within the olfactory cortex (1). The incidence of olfactory dysfunction after head injury was reported to be approximately 13%, with regards to the site and nature of injury: injury involving a fracture of base of the skull and presence of frontal hematoma/hemorrhage is associated with a higher probability of ensuing olfactory dysfunction (2).

Extensive literature shown the existence of multiple etiologies underlying olfaction disorders. In addition to head and facial injuries, viral/bacterial inflammatory and neurological conditions such as neurodegenerative diseases are common etiologies reported in current literature (13). Irregularities in the function of the olfactory system are also often caused by the intense impact of numerous aggressive environmental factors associated with the nature of the work performed or other long-term exposure, including heavy metals (such as iron, cadmium, chromium) and by vapors of many organic and inorganic chemicals (acetone, benzene, menthol) which result in harmful effects on the olfactory organs (14, 15). Iatrogenic diseases and the use of recreational drugs additionally constitute an important group of factors causing dysfunction or loss of smell. In healthy persons, olfactory dysfunction becomes increasingly common with advancing age (13).

Olfactory deficits often go undiscovered after the inciting event, due to accompanying neurologic and orthopedic injuries which require immediate stabilization and management, as well as cognitive impairment which limits a patient's own recognition of olfactory deficits. The sense of smell is particularly important in recognition of danger, in interpersonal communication, and in eating and drinking. Impairment in patients may lead to a decreased QoL and limitations in their professional work, as reported in patients within the studied group, implying the need for a routine examination of the sense of smell in patients after head trauma. After the initial evaluation including a comprehensive history and physical exam, and CT/MRI scanning to determine to site of injury, quantitative olfactory testing could be used to verify the presence of olfactory deficit and assess its degree of severity.

### Olfactometric Profiles of Tested Subjects

In recent years, multiple tests of olfaction have been introduced in otorhinolaryngology and neurology. Subjective examinations of olfaction remain the conventional tool in the investigation of olfactory deficit in routine neurological assessment. Objective examinations, which reveal OERPs arising from the anterior-central parts of the insula, the parainsular cortex, and the superior temporal sulcus, are not usually employed in routine clinical assessment of anosmia (16). Our findings indicate that without an objective assessment, the accuracy of a patient's olfaction cannot be definitively established in patients with post-traumatic anosmia.

The loss of OERPs indicates a lesion proximal to these limbic regions, such as damage to olfactory nerves, bulbs, and tracts. Damage to the central (perceptive) olfactory system can be established when there is a reduction in amplitude or absence of OERPs in addition to the impairment in olfactory perception and identification in the subjective examination, as presented in 12 patients (31.6%) in the present study. A wide range of amplitudes of registered potentials for tested subjects were observed during the objective olfactory examination. The amplitudes of responses were dependent on factors such as the electrical resistance of the skin, the speed of the processes of adaptation to odor and the differences in the anatomical structure of the nasal cavity as the center conducting the scent factor to the olfactory epithelium.

Loss of olfactory function without the loss of OERPs suggests lesions to the distal parts of the olfactory brain such as sinonasal tract or peripheral nerve disruption. Apart from peripheral injury, these findings could be attributed to impaired cognition secondary to brain injury or malingering for which financial incentives might exist to increase the apparent debility resulting from the injury. In another regard, the recorded responses transmitted via olfactory pathway without the awareness of olfactory sensation by the patient may be a reflection of the regenerative process of the epithelium or nerve fiber. Such situation can be observed in cognitive potentials mismatch negativity. Similar observations were previously described, one example being the observation of the recorded the cognitive potential, the mismatch negativity wave, in the dyslexic children before the behavioral changes (discrimination of speech sounds) (17, 18).

Different degrees of head injuries may be complicated by post-traumatic chemosensory dysfunction. The severity and duration of post-traumatic amnesia has been reported as predictive factors. The incidence of olfactory impairment following mild, moderate, and severe TBI has been reported to be approximately up to 16, 19, and 30%, respectively (1, 12). The result of olfaction assessment help to verify the degree of central nervous system damage. The differences in subjective and objective olfaction tests in our study highlight the utility of objective testing in establishing the extent of the patient's condition, in detecting malinger and helping to establish an accurate disability compensation, and in monitoring functional changes over time.

### Limitations and Future Perspectives

The present study has a number of limitations. Firstly, our study can be enhanced by a further evaluation of testing results comparing subjects who are cognitively impaired to those who aren't. The level of TBI severity and frequency among study subjects should be examined in more detail as this may be a predictor of olfactory dysfunction. In our study, TBI was assessed through routine neurological examinations upon the patient's presentation to the clinic. This can be enhanced by the use of a standardized, validated measure for each patient in order to enhance reproducibility and reduce difficulties in replication studies. Additional clinical determinants of injury severity, as the presence and duration of loss of consciousness, should be taken into consideration. The clinical impact of uniformly performing both tests on diagnosis and treatment should also be taken into account in future research. It should be noted that this study was conducted in a single medical hospital. As not all patients after trauma view anosmia as a problem, it is often under-reported, and this contributes to our small sample size in the present study. Replication of the analyses on different, larger cohorts is necessary to further validate this methodology and to establish a more detailed conclusion.

## Conclusion and Implications for Clinical Practice

The common expression “CN II-XII intact” reflects how olfactory testing is commonly disregarded in clinical evaluation. The possibility of a diminished QoL and an increased risk for personal injury in the patient's everyday life is in-turn underevaluated and overlooked. Differences in subjective and objective measures of olfaction in this study describes a complexity in the clinical diagnosis of post-traumatic olfactory dysfunction. Our findings indicate the utility of objective examinations in the diagnosis of anosmia and hyposmia in addition to routine subjective testing. Objective examinations are useful particularly in post-traumatic situations in which the patients' level of cognition may be impaired or when subjects may be exaggerating their olfactory defects for a secondary gain. In addition to the diagnosis of olfactory impairment, the use of both objective and subjective examinations may help to differentiate the central or peripheral location of damage to the olfactory system, and help guide treatment and monitor changes in olfactory function over time. Beyond neurotrauma, olfactory impairment may be a pre-clinical sign of a diverse array of medical conditions, from neurodegenerative diseases to the severe acute respiratory syndrome-coronavirus-2 (SARS-CoV-2) infection, in which proper evaluation is implicated for a comprehensive intervention (19). Objective testing in olfaction may be particularly useful in patients with compromised complex cognitive functions or attention levels. An algorithm in the evaluation of patients with olfactory impairment has been suggested to help guide physicians in differentiating the complex underlying etiologies of olfactory dysfunction (20). Results from our study indicate the application of both quantitative subjective with the objective examinations in the facilitation of this method.

## Data Availability Statement

The datasets generated for this study are available on request to the corresponding author.

## Author Contributions

All authors listed have made a substantial, direct and intellectual contribution to the work, and approved it for publication.

## Conflict of Interest

The authors declare that the research was conducted in the absence of any commercial or financial relationships that could be construed as a potential conflict of interest.
